# A prospective investigation of oral contraceptive use and breast cancer mortality: findings from the Swedish women’s lifestyle and health cohort

**DOI:** 10.1186/s12885-019-5985-6

**Published:** 2019-08-14

**Authors:** Ula Nur, Darline El Reda, Dana Hashim, Elisabete Weiderpass

**Affiliations:** 1 Department of Public Health, College of Health Sciences, QU Health, Qatar University, P.O. Box, 2713, Doha, Qatar; 2 Michigan Medical Advantage Group, Ann Arbor, MI USA; 3 College of Human Medicine, Division of Public Health, Michigan State University, East Lansing, MI USA; 4 Cancer Registry of Norway, Institute of Population-Based Cancer Research, Oslo, Norway; 5 Institute of Basic Medical Sciences, University of Oslo, Oslo, Norway; 6 International Agency for Research on Cancer, Lyon, France

**Keywords:** Oral contraceptives, Breast cancer, Survival, Multiple imputation, Hormone receptor status, Stage

## Abstract

**Background:**

The association between oral contraceptive (OC) use and long-term mortality remains uncertain and previous studies have reported conflicting findings. We aim to assess the long-term impact of OC use on all-cause and cancer-specific mortality.

**Methods:**

Out of 49,259 participants, we analysed data on 2120 (4.3%) women diagnosed with first primary breast cancer between 1993 and 2012, in the Swedish Women’s Lifestyle and Health Study. Kaplan–Meier plots were used to graph the hazard of mortality in association with oral contraceptives use, stage of disease and hormone receptors status at diagnosis. Cox proportional hazard model were used to estimate hazard ratios (HR) between OC use and all-cause mortality. The same association was studied for breast cancer-specific mortality by modelling the log cumulative mortality risk, adjusting for clinical stage at diagnosis, hormone receptor status, body mass index and smoking.

**Results:**

Among 2120 women with breast cancer, 1268 (84%) reported ever use of OC and 254 died within 10 years of diagnosis. The risk of death for OC ever-users relative to never-users was: HR = 1.13 (95% CI: 0.66–1.94) for all-cause mortality and HR = 1.29 (95% CI: 0.53–3.18) for breast cancer-specific mortality. A high percentage of women (42.9%) were diagnosed at early stage disease (stage I).

**Conclusions:**

Among women with primary breast cancer, OC ever-users compared to never- users did not have a higher all-cause or breast cancer specific-mortality, after the adjustment of risk factors.

**Electronic supplementary material:**

The online version of this article (10.1186/s12885-019-5985-6) contains supplementary material, which is available to authorized users.

## Background

Breast cancer is the most common cancer in women worldwide, with an estimated 1.7 million new cases diagnosed in 2012; representing about 12% of all new cancer cases and 25% of all cancers in women [1]. Across all countries in Europe, the breast is the leading cancer site in women. Western Europe has the second highest incidence rate of breast cancer worldwide; in Sweden, the age-adjusted incidence rate is approximately 81.4/100,000 women [2, 3].

The association between oral contraceptive (OC) use and the subsequent risk of breast cancer has been well-studied. The International Agency for Research on Cancer published a monograph in 2007, in which a scientific specialist review panel agreed that there was sufficient evidence for an association between OC use and breast cancer risk in humans [4]. However, this assessment found inconsistent results for women who had ever used OC versus never-users. The increased risk was only noted for women who were current or recent OC users, particularly those who were less than 35 years of age at diagnosis [4].

The more recent cohort studies which have examined the association between ever OC use and all-cause mortality or breast cancer-specific mortality among women with breast cancer, continue to report inconsistent results [5–11]. A number of cohort studies reported no association [5, 6, 9–11], while other cohort studies reported that OC use slightly reduces the risk of all-cause mortality among women with breast cancer [7, 8]. For breast cancer-specific mortality, the majority of studies reported no association with OC use [6–8, 10, 12]. However, findings from the largest cohort to date, The Nurses’ Health Study [5], which included 121,577 women, found that OC use is associated with increased rates of death due to breast cancer for women who have used OC for 5 years or more compared to never-users (hazard ratio (HR) = 1.26; 95% confidence interval (CI): 1.09 to 1.46.

OC is one of the most common used contraceptive methods in Western Europe with an estimated 43.5% of use among women of reproductive age [13]. In Sweden in particular, an estimated 65 to 88% of women currently use or have used OCs [14–17]. Yet, studies on the association between OC use and mortality among women with breast cancer in Sweden are not available. Given the public health implications and lack of consistent findings, further study on the relationship between OC use and breast cancer mortality is warranted.

The Swedish Women’s Lifestyle and Health (WLH) cohort was designed to study the role of hormonal contraceptives in relation to breast cancer in Norway and Sweden, populations with high prevalence of OC use and high breast cancer incidence rates [14, 17]. Using this population-based sample of Swedish women, we examine the association between OC use on both all-cause and breast cancer-specific mortality among women diagnosed with breast cancer between 1993 and 2013.

## Methods

### Study population

The WLH was designed to prospectively investigate the association between lifestyle factors and cancer and cardiovascular disease outcomes in women [14]. A total of 96,000 women born between 1943 and 1962 residing in the Uppsala Health Care Region, who met the initial inclusion criteria, were randomly selected from the Swedish Population Registry and invited to participate via a mailed questionnaire. During 1991–1992, 49,259 (51%) women responded to the baseline questionnaire (Q1) and thus recruited into the WLH cohort. The questionnaires captured data on a variety of demographic, lifestyle and health factors, including oral contraceptive use (ever-used vs. never-used), height, weight and smoking. Ethical approval was obtained from the Swedish Data Inspection Board, the Regional Ethnical Committee of Uppsala University, and the Ethical Committee of the Karolinska Institutet.

### Data linkage

The cohort data was linked to the Swedish Cancer Registry database using personal identification number (PIN) to identify all cases of breast cancer among participants. The registry utilised three types of information: 1) Individual patient demographics: sex, age, place of residence; 2) Medical data: tumour site, histological type, Tumour-Node-Metastasis (TNM) (6th edition) stage, basis of diagnosis, date of diagnosis, hormone receptors (estragon and progesterone) and disease grade; 3) Follow-up data: date of death, cause of death, date of emigration. The Population Register is the civil registration of vital events, such as place and date of death, burial site, and marriages for people born in Sweden or emigrate in/out of Sweden. Information on death and emigration were extracted from the Population Register using PINs.

### Case assessment and risk factors

All women diagnosed with an invasive, primary malignant neoplasm of the breast (International Classification of Diseases, tenth revision [13] (ICD-10)), from enrolment until 31 December 2012 were considered for analysis. We excluded all cases with breast cancer or a second primary malignancies (*n* = 24) at the time of recruitment to the study. Follow-up was calculated from the date of breast cancer diagnosis to emigration, death or study end point of 31 December 2013. A final dataset comprised of 2120 cases of breast cancer was analysed. Body mass index (BMI) was calculated as follows: weight (kg) divided by the square of height, and the following World Health Organization categories for BMI were used: underweight, BMI < 18.5; normal, 18.5 ≥ BMI ≤ 24.9; overweight, 25 ≥ BMI ≤ 29.9; obesity, BMI ≥ 30. Only 2% (*n* = 42) of the study population were underweight, thus we combined the underweight and normal category. Smoking at Q1 was collected in three categories; current smokers, former smokers, never smokers. Each patient was assigned to one of four categories of (ER/PR) based on their ER (estrogen) and PR (progesterone) receptors status (ER+/PR+, ER−/PR+, ER+/PR-, ER−/PR-). Data on TNM stage at diagnosis was converted into the clinical five-level categories: 0, I, II, III & IV. The number of patients diagnosed at more advanced stages were low and therefore, stages II, III and IV were combined.

### Statistical analysis

Person-time was calculated from the date of diagnosis with breast cancer to death date or the last date of follow up. Follow-up time was censored 10 years after diagnosis. Kaplan–Meier plots were used to graph the hazard of mortality in association with OC use, and on potential confounders, where missing values were considered as a distinct category. Smoothed hazard plots were used to graph mortality from breast cancer in association with OC use and stage of disease. We estimated Hazard Ratios (HR) and 95% confidence intervals (CIs) for the association between OC use and the risk of death from all causes (all-cause mortality) among women with breast cancer, using Cox proportional hazards model and adjusting for age at diagnosis, BMI, hormone receptors status (ER/PR), stage of disease at diagnosis, and smoking. Schoenfeld residuals were used to assess the proportional hazards assumption. When the proportional hazards hypothesis was not satisfied, we introduced a time function to model estimated time-varying HRs.

Cancer-specific mortality, generally known as ‘excess’ mortality due to cancer, was modelled as the difference between all-cause mortality (observed) experienced by cancer patients and the expected (background) mortality of a comparable group from the general population. This approach enabled population-level cancer-specific mortality to be estimated in the absence of detailed information on the cause of death. The background mortality was derived from population life-tables that were constructed by single year of age (0–99 years) and single calendar year (1993–2013) and sex, for the entire population of Sweden. Cancer-specific mortality was modelled on the log cumulative hazard scale in a flexible parametric framework [18, 19] using the stpm2 [20] command in Stata version 14 [21], to predict the effect of oral contraceptive use on breast cancer-specific mortality after adjusting for age at diagnosis, BMI, hormone receptors status (ER/PR), stage of disease at diagnosis, and smoking. Data were incomplete for OC use and three of the predictor variables.

The simplest way to analyse data with incomplete variables is to exclude all records (cases) that are incomplete. This method is known as the complete-case analysis. Analysis of complete records may yield results that could be substantially different from those that would be obtained if complete information were collected on all variables. Multiple imputation [22, 23] was used to account for the incompleteness on OC, BMI, stage, and ER/PR (Table 1), under the assumption that data were missing at random (MAR). For each of the four possible incomplete variables (OC use (*n* = 615; 29.0% missing), stage (*n* = 495; 23.4% missing), BMI (*n* = 88;4.15% missing), or ER/PR receptor status (*n* = 415;19.6% missing), we derived imputation models, that included the remaining three incomplete variables in addition to the complete variables for which no data were missing: age, smoking, vital status and the cumulative survival time (Nelson Aalen) [24]. We created 10 ‘completed’ data sets from the ‘observed’ and the ‘imputed’ values. Analysis models were fit for each completed dataset and results were combined under Rubin’s rules [22]. Sensitivity analyses were performed to assess the robustness of results against departure from the MAR assumption (results not shown). All analyses were carried out in STATA version 14 [21].

**Table 1:**
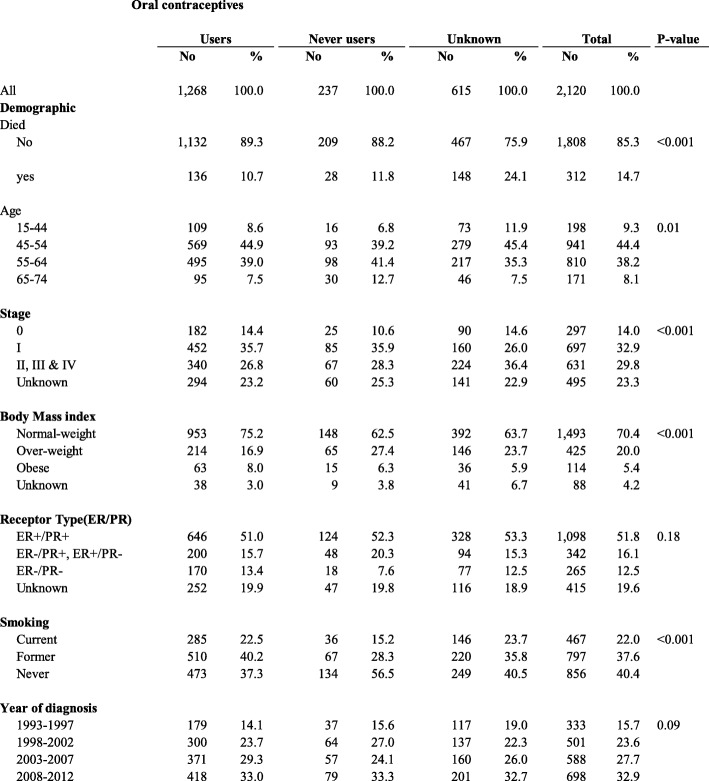
Characteristics of women with breast cancer, recruited to The Swedish Women’s Lifestyle and Health (WLH) (*N* = 2120), by oral contraceptive use, 1993–2013

## Results

### Demographics

Baseline characteristics for the population of 2120 Swedish women diagnosed with breast cancer before enrolment are summarised in Table 1. The median follow-up was 7.63 years. The mean age of breast cancer diagnosis for this cohort was 55 years; 45% of women were diagnosed within the age range of 45–54 years. Among women with TNM stage, 42.9% were diagnosed with stage I disease, 33.9% with stage II and 3.8% with stage III and IV; therefore stages II, III & IV were combined for subsequent analyses. Only 52 (2.5%) women were identified with hormone receptor status ER−/PR+. This receptor type was combined with ER+/PR- for analysis. Among women who reported OC use, 84.2% (1268) reported ever use. Data for some women were incomplete for the following variables: OC use (615 women, 29.1%), stage at diagnosis (495 women, 23.4%) and receptor type (415 women, 19.6%) and BMI (88 women, 4.15%). The distribution of age and stage at diagnosis was similar for ever and never-users of OC. A higher percentage of never-users of OC were above normal weight (37.5%) compared to 24.9% of OC ever-users.

For a proportion of the 2120 women with breast cancer, data were missing on OC use (615, 29.0%), stage (495, 23.4), BMI (88, 4.15) and ER/PR receptor status (415, 19.6%). Data on OC use was missing more often on women diagnosed at (II, III & IV) stage of disease, who were Normal-weight, and those diagnosed between 15 and 44 years of age (Table 1).

### All-cause mortality

**Fig. 1:**
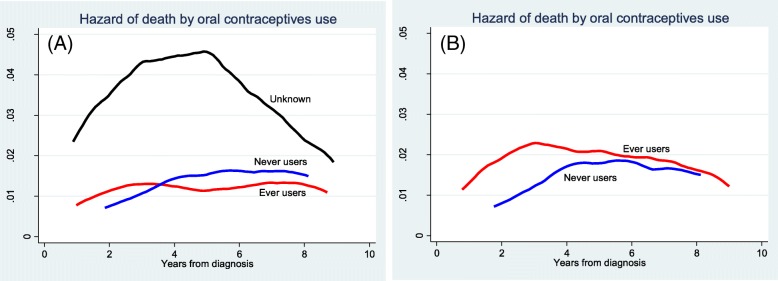
Mortality risk from all causes, up to 10 years after diagnosis, by oral contraceptive use among women diagnosed with breast cancer in the Swedish women’s lifestyle and health cohort. **a** without imputation of missing values for oral contraceptive use **b** after imputation of missing values for oral contraceptive use

Patients with unknown OC use had the highest mortality risk compared to ever and never-users. OC ever-users had slightly higher mortality risk up to almost 3 years after diagnosis, however mortality risk for never-users increased and became higher than OC ever-users by 3.5 years after diagnosis (Fig. 1a).

Because of the incompleteness of data on OC use, and three risk factors; BMI, stage, and ER/PR (Table 1), only 1014 (47.83%) of the study population could be analysed in the multivariable model using the complete-case analysis (Additional file 1).

**Fig. 2:**
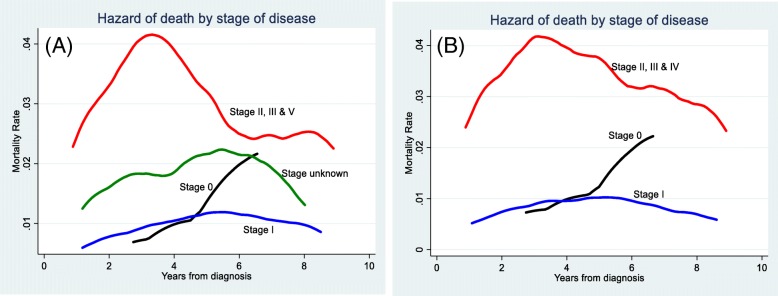
Mortality risk from all causes, up to 10 years after diagnosis, by stage of disease among women diagnosed with breast cancer in the Swedish women’s lifestyle and health cohort. **a** without imputation of missing values for stage of cancer **b** after imputation of missing values for stage of cancer

**Fig. 3:**
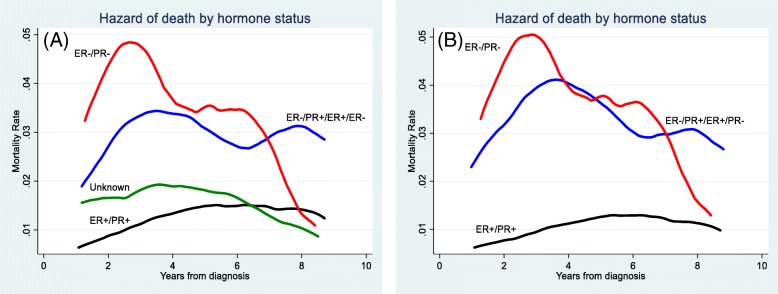
Mortality risk from all causes, up to 10 years after diagnosis, by hormone status among women diagnosed with breast cancer in the Swedish women’s lifestyle and health cohort. **a** without imputation of missing values for hormone status **b** after imputation of missing values for hormone status

After handling the unknowns for OC using multiple imputation, the mortality risk for OC ever-users compared to never-users was higher throughout the follow-up period. Women diagnosed with breast cancer at stages II, III & IV had higher risk of death up to at least 10 years after diagnosis; before and after multiple imputation (Fig. 2a and b). However, mortality risk for the same period was much lower and with smaller difference for those diagnosed at stage 0 and I (Fig. 2a and b). Risk of death by hormone receptor status at diagnosis varied considerably up to 4 years after diagnosis, for women diagnosed with hormone receptor status ER−/PR- (higher risk) than women with ER-PR+/ER + PR- or ER + PR+ (Fig. 3a and b).

All-cause mortality did not significantly differ between OC ever-users and never-users (HR = 1.13, 95% CI: 0.66–1.94) after adjusting for covariates (Table 2).

**Table 2:**
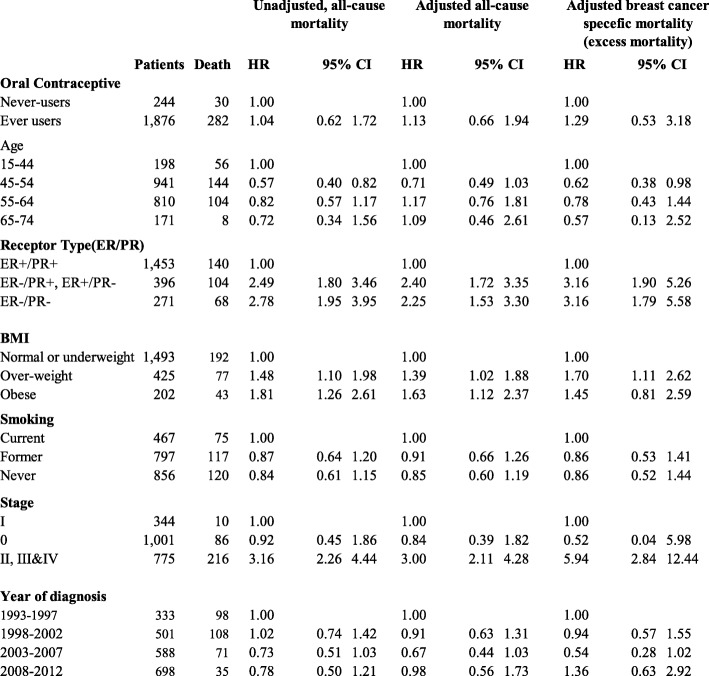
Hazard ratios of mortality (unadjusted and adjusted), among women with breast cancer, in relation to OC, among 2120 women recruited to the WLH study, 1993–2013

*Models were adjusted for age at diagnosis, hormone receptor status, body mass index, smoking, stage at diagnosis and year of diagnosis

### Breast-cancer mortality

When the survival analysis was restricted to breast-cancer specific mortality only, HRs of similar magnitude and significance to all-cause mortality were observed (HR = 1.29; 95% CI: 0.53–3.18). (Table x).

OC ever-users had a higher breast-cancer specific mortality risk compared to OC never-users (HR = 1.29, 95% CI: 0.53–3.18). This non-significant effect of OC use was higher than that observed for all-cause mortality (Table 2).

## Discussion

We studied the risk of OC use on all-cause and breast cancer-specific mortality among 2120 Swedish women enrolled in a population-based cohort (WLH) and subsequently diagnosed with breast cancer between 1992 and 2012. Women who were ever-users of OC did not have a higher all-cause or breast cancer-specific mortality as compared to never-users.

Clinical stage at diagnosis, hormone receptor status, BMI, year of diagnosis, smoking, and age were strong confounders for the association between OC use and mortality. The relationship between OC and mortality would have been underestimated without accounting for these variables. Our findings are in alignment with the findings of a number of recently published studies which have explored OC use and mortality and reported hazard ratios of similar magnitude [6, 10, 25]. Results were also similar to those of the Nurses’ Health Study, which examined mortality in association with OC use up to 36 years after diagnosis with breast cancer [5]. However, a retrospective study of a population-based cohort of 4816 women found that ‘estrogen-progestin’ OC use increased the risk of breast cancer mortality and all-cause mortality (HR: 1.61; 95% CI: 1.14, 2.28) and (HR: 1.83; 95% CI: 1.30, 2.57), respectively [25]. It is possible that differences in surveillance may bias the relationship between OC use and breast cancer [17], with women receiving OC more likely to attend breast cancer screening and wellness visits, thereby having lower mortality rates. This potential bias is reduced in our study, as all women in Sweden have undergone similar medical surveillance and have the same access to medical care. The finding related to hormone receptors (ER−/PR+ or ER+/PR-) and increased risk of breast cancer mortality was also consistent with other studies [26, 27].

The WLH cohort was designed to study the role of hormonal contraceptives in relation to breast cancer in Norway and Sweden; populations with a high prevalence of OC use and high breast cancer incidence rates [14, 17]. Before 1976, OC preparations were likely to contain high doses of estrogens and/or progestins, which likely applies to women in this birth cohort (born between 1943 and 1962) [4, 15]. The observation of a small non-significant increased breast cancer risk among women who were current/recent users of combined OCs (for example, those containing an estrogen and a progestin) is compatible with the “estrogen plus progestin” theory of breast cancer development. This theory implies that the combination of hormones induces more cell divisions than estrogen alone [28]. Use of combined OCs directly increases levels of estrogen as well as progestogens, whereas progestin-only pills only increase levels of progestogens without directly raising estrogen levels. Estimates for breast cancer risk among progestin-only pill users in this study could not be estimated due to small sample sizes. Although present-day OC hormonal formulations may differ from those used by this cohort of women [14], it is nevertheless re-assuring that this cohorts’ prior use of OC during their reproductive years is not likely to increase their risk of mortality.

The generalizability of our findings may be limited. Our cohort consisted of Swedish women of the same generation/similar birth cohort, who were well-educated (over 80% with high school education). OC use was self-reported prior to breast cancer diagnosis, however estimates for these women were similar with what was reported for the larger baseline cohort [14]. Information on the use of hormone replacement therapy (HRT) was collected at baseline (1991–92), and follow-up (2003). The majority of baseline cohort (87.52%) and follow-up cohort (88.29%) were pre-menopausal [14]. Only 5% of cohort participants developed breast cancer after recruitment to the cohort used HRT [17]. HRT has been associated with a better prognosis of hormone receptor positive breast cancer [29], thus HRT was excluded from analysis. Missing data are a common problem in large surveys, for which data are collected using extensive mailed questionnaires such as The WLH. High levels of incompleteness of OC use and the strong predictors of mortality such as stage of disease, hormone receptor type and BMI, complicate the analysis and can lead to biased results [23, 24, 30]. We applied the method of multiple imputation to account for missing data, including all relevant cancer cases in the multivariable analysis. Imputation models included all the variables in the analysis model, the cumulative survival time (Nelson Aalen) [24] and vital status [24]. This method is only valid under the assumption that data is missing at random (MAR), which can never be validated with absolute certainty. However, including all predictors of missingness and the outcome variables in the imputation model improved the validity of the assumption of MAR.

A key strength of this study is the use of reliable and validated data from a population-based cohort with almost complete follow-up for all participants up to 10 years after diagnosis, linked to the Swedish Cancer Registry, with high levels of completeness [31]. We were also able to adjust for the potential confounding effect of stage at diagnosis and hormone receptor status (estrogen and progesterone) in the estimated effect of OC use on mortality. Our ability to adjust for receptor type is important given that ER- tumours have been demonstrated to have a worse prognosis compared to ER+ tumours [27]. The association between ever-use of OC and estrogen receptor-negative (ER-) breast cancer as compared with ER+ cancer, however, is less clear; with a number of past studies reporting strong associations [32–36], and other studies concluding little or no difference [37–42]. The prospective design of this study also increases our confidence for a lack of causal association found between OC use and breast-cancer-specific and all-cause mortality.

Another strength of this study is that use or non-use of OCs is not likely to have influenced the timing of receipt of a breast cancer diagnosis given that annual physical examinations (or ‘check-ups’) are not regularly performed or required in Sweden. Breast cancer screening initiatives are organized outside medical doctors’ offices, and screening tests are carried out by nurses who refer patients for follow-up with physicians when a pre-malignant or malignant lesion is suspected.

## Conclusion

Our results suggest that women with breast cancer who were ever-users of OC, as compared to never-users of OC, did not experience a higher all-cause or breast cancer-specific mortality, after the adjustment of risk factors. Our results relate to OC use at the study time, and that we cannot rule out that current OCs may show a different association. More research is needed on duration of contraceptive use, and biological underpinnings behind OC use cessation in relation to breast cancer mortality to clarify and support this evidence.

## Additional file

Additional file 1: Hazard ratios of mortality (unadjusted and adjusted), among women with breast cancer, in relation to OC, among 1014 women recruited to the WLH study, 1993–2013, using complete cases. (DOCX 25 kb)

## Data Availability

Data is available upon request using a signed application from the Karolinska institutet website.

## Abbreviations

BMI: Body mass index
ER: Estrogen
ER/PR: Hormone receptors status
HR: Hazard ratios
OC: Oral contraceptives
PR: Progesterone
WLH: The Swedish Women’s Lifestyle and Health cohort

## References

[1] Ferlay J, Soerjomataram I, Ervik M, Dikshit R, Mathers C, Rebelo M, Parkin DM, Forman D, Bray F (2014). GLOBOCAN 2012 v1.0, Cancer incidence and mortality worldwide: IARC CancerBase no. 11.

[2] Ferlay J, Steliarova-Foucher E, Lortet-Tieulent J, Rosso S, Coebergh JW, Comber H, Forman D, Bray F (2013). Cancer incidence and mortality patterns in Europe: estimates for 40 countries in 2012. Eur J Cancer.

[3] Gierisch JM, Coeytaux RR, Urrutia RP, Havrilesky LJ, Moorman PG, Lowery WJ, Dinan M, McBroom AJ, Hasselblad V, Sanders GD (2013). Oral contraceptive use and risk of breast, cervical, colorectal, and endometrial cancers: a systematic review. Cancer Epidemiol Biomarkers Prev.

[4] IARC: Combined estrogen-progestogen contraceptives and combined estrogen-progestogen menopausal therapy. In: IARC Monographs on the Evaluation of Carcinogenic Risks to Humans*.* 91. Lyon: International Agency for Research on Cancer; 2007: 1–528. PMC4781221 18756632

[5] Charlton BM, Rich-Edwards JW, Colditz GA, Missmer SA, Rosner BA, Hankinson SE, Speizer FE, Michels KB (2014). Oral contraceptive use and mortality after 36 years of follow-up in the Nurses’ health study: prospective cohort study. Bmj.

[6] Lu Y, Ma H, Malone KE, Norman SA, Sullivan-Halley J, Strom BL, Simon MS, Marchbanks PA, McDonald JA, West DW (2011). Oral contraceptive use and survival in women with invasive breast cancer. Cancer Epidemiol Biomarkers Prev.

[7] Vessey M, Yeates D, Flynn S (2010). Factors affecting mortality in a large cohort study with special reference to oral contraceptive use. Contraception.

[8] Hannaford PC, Iversen L, Macfarlane TV, Elliott AM, Angus V, Lee AJ (2010). Mortality among contraceptive pill users: cohort evidence from Royal College of general Practitioners’ Oral contraception study. Bmj.

[9] Phillips KA, Milne RL, West DW, Goodwin PJ, Giles GG, Chang ET, Figueiredo JC, Friedlander ML, Keegan TH, Glendon G (2009). Prediagnosis reproductive factors and all-cause mortality for women with breast cancer in the breast cancer family registry. Cancer Epidemiol Biomarkers Prev.

[10] Trivers KF, Gammon MD, Abrahamson PE, Lund MJ, Flagg EW, Moorman PG, Kaufman JS, Cai J, Porter PL, Brinton LA (2007). Oral contraceptives and survival in breast cancer patients aged 20 to 54 years. Cancer Epidemiol Biomarkers Prev.

[11] Graff-Iversen S, Hammar N, Thelle DS, Tonstad S (2006). Use of oral contraceptives and mortality during 14 years’ follow-up of Norwegian women. Scand J Public Health.

[12] Wingo PA, Austin H, Marchbanks PA, Whiteman MK, Hsia J, Mandel MG, Peterson HB, Ory HW (2007). Oral contraceptives and the risk of death from breast cancer. Obstet Gynecol.

[13] United Nations Population Division. World Contraceptive Use 2005. New York: Department of Economics and Social Affairs; 2006.

[14] Roswall Nina, Sandin Sven, Adami Hans-Olov, Weiderpass Elisabete (2015). Cohort Profile: The Swedish Women’s Lifestyle and Health cohort. International Journal of Epidemiology.

[15] Ranstam J, Olsson H (1993). Oral contraceptive use among young women in southern Sweden. J Epidemiol Community Health.

[16] Kopp Kallner H, Thunell L, Brynhildsen J, Lindeberg M, Gemzell Danielsson K (2015). Use of contraception and attitudes towards contraceptive use in Swedish women--a Nationwide survey. PLoS One.

[17] Kumle M, Weiderpass E, Braaten T, Persson I, Adami HO, Lund E (2002). Use of oral contraceptives and breast cancer risk: the Norwegian-Swedish Women's lifestyle and health cohort study. Cancer Epidemiol Biomarkers Prev.

[18] Nelson CP, Lambert PC, Squire IB, Jones DR (2007). Flexible parametric models for relative survival, with application in coronary heart disease. Stat Med.

[19] Royston P, Sauerbrei W (2007). Multivariable modeling with cubic regression splines: a principled approach. Stata J.

[20] Lambert PC, Royston P (2009). Further development of flexible parametric models for survival analysis. Stata J.

[21] Statacorp (2014). STATA statistical software.

[22] Rubin DB (1987). Multiple imputation for nonresponse in surveys.

[23] Nur U, Shack LG, Rachet B, Carpenter JR, Coleman MP (2010). Modelling relative survival in the presence of incomplete data: a tutorial. Int J Epidemiol.

[24] Falcaro M, Nur U, Rachet B, Carpenter JR (2015). Estimating excess hazard ratios and net survival when covariate data are missing: strategies for multiple imputation. Epidemiology.

[25] Samson ME, Adams SA, Mulatya CM, Zhang J, Bennett CL, Hebert J, Steck SE (2017). Types of oral contraceptives and breast cancer survival among women enrolled in Medicaid: a competing-risk model. Maturitas.

[26] El Saghir NS, Assi HA, Jaber SM, Khoury KE, Nachef Z, Mikdashi HF, El-Asmar NS, Eid TA (2014). Outcome of breast Cancer patients treated outside of clinical trials. J Cancer.

[27] Chlebowski RT, Chen Z, Anderson GL, Rohan T, Aragaki A, Lane D, Dolan NC, Paskett ED, McTiernan A, Hubbell FA (2005). Ethnicity and breast cancer: factors influencing differences in incidence and outcome. J Natl Cancer Inst.

[28] Chlebowski RT, Kuller LH, Prentice RL, Stefanick ML, Manson JE, Gass M, Aragaki AK, Ockene JK, Lane DS, Sarto GE (2009). Breast cancer after use of estrogen plus progestin in postmenopausal women. N Engl J Med.

[29] Rauh C, Schuetz F, Rack B, Stickeler E, Klar M, Orlowska-Volk M, Windfuhr-Blum M, Heil J, Rom J, Sohn C (2015). Hormone therapy and its effect on the prognosis in breast Cancer patients. Geburtshilfe Frauenheilkd.

[30] Schottenfeld D, Winawer SJ: Cancers of the large intestine. In: Cancer Epidemiology and Prevention. 2nd. Edited by D S, Jr FJ. Oxford: Oxford University Press; 1996: 813–840.

[31] Barlow L, Westergren K, Holmberg L, Talback M (2009). The completeness of the Swedish Cancer register: a sample survey for year 1998. Acta Oncol.

[32] Sweeney C, Giuliano AR, Baumgartner KB, Byers T, Herrick JS, Edwards SL, Slattery ML (2007). Oral, injected and implanted contraceptives and breast cancer risk among U.S. Hispanic and non-Hispanic white women. Int J Cancer.

[33] Althuis MD, Brogan DD, Coates RJ, Daling JR, Gammon MD, Malone KE, Schoenberg JB, Brinton LA (2003). Breast cancers among very young premenopausal women (United States). Cancer Causes Control.

[34] Cooper JA, Rohan TE, Cant EL, Horsfall DJ, Tilley WD (1989). Risk factors for breast cancer by oestrogen receptor status: a population-based case-control study. Br J Cancer.

[35] Dolle JM, Daling JR, White E, Brinton LA, Doody DR, Porter PL, Malone KE (2009). Risk factors for triple-negative breast cancer in women under the age of 45 years. Cancer Epidemiol Biomarkers Prev.

[36] Ma H, Bernstein L, Ross RK, Ursin G (2006). Hormone-related risk factors for breast cancer in women under age 50 years by estrogen and progesterone receptor status: results from a case-control and a case-case comparison. Breast Cancer Research.

[37] Cotterchio M, Kreiger N, Theis B, Sloan M, Bahl S (2003). Hormonal factors and the risk of breast cancer according to estrogen- and progesterone-receptor subgroup. Cancer Epidemiol Biomarkers Prev.

[38] Huang WY, Newman B, Millikan RC, Schell MJ, Hulka BS, Moorman PG (2000). Hormone-related factors and risk of breast cancer in relation to estrogen receptor and progesterone receptor status. Am J Epidemiol.

[39] McCredie MR, Dite GS, Southey MC, Venter DJ, Giles GG, Hopper JL (2003). Risk factors for breast cancer in young women by oestrogen receptor and progesterone receptor status. Br J Cancer.

[40] McTiernan A, Thomas DB, Johnson LK, Roseman D (1986). Risk factors for estrogen receptor-rich and estrogen receptor-poor breast cancers. J Natl Cancer Inst.

[41] Rosenberg L, Zhang Y, Coogan PF, Strom BL, Palmer JR (2009). A case-control study of oral contraceptive use and incident breast cancer. Am J Epidemiol.

[42] Stanford JL, Szklo M, Boring CC, Brinton LA, Diamond EA, Greenberg RS, Hoover RN (1987). A case-control study of breast cancer stratified by estrogen receptor status. Am J Epidemiol.

