# Supplementary material for: A prospective investigation of oral contraceptive use and breast cancer mortality: findings from the Swedish women’s lifestyle and health cohort
Source: BMC Cancer. 2019 Aug 14;19:807. doi: 10.1186/s12885-019-5985-6 (PMC6694621; doi:10.1186/s12885-019-5985-6)
Supplement: Supplementary file 1 — Hazard ratios of mortality (unadjusted and adjusted), among women with breast cancer, in relation to OC, among 1014 women recruited to the WLH study, 1993–2013, using complete cases. (DOCX 25 kb) [file 12885_2019_5985_MOESM1_ESM.docx]

*Models were adjusted for age at diagnosis, hormone receptor status, body mass index, smoking, stage at diagnosis and year of diagnosis
